# S-Carboxymethyl Cysteine Protects against Oxidative Stress and Mitochondrial Impairment in a Parkinson’s Disease In Vitro Model

**DOI:** 10.3390/biomedicines9101467

**Published:** 2021-10-14

**Authors:** Mariano Catanesi, Laura Brandolini, Michele d’Angelo, Maria Grazia Tupone, Elisabetta Benedetti, Margherita Alfonsetti, Massimiliano Quintiliani, Maddalena Fratelli, Daniela Iaconis, Annamaria Cimini, Vanessa Castelli, Marcello Allegretti

**Affiliations:** 1 Department of Life, Health and Environmental Sciences, University of L’Aquila, 67100 L’Aquila, Italy; mcatanesi@unite.it (M.C.); michele.dangelo@univaq.it (M.d.); mariagrazia.tupone@univaq.it (M.G.T.); elisabetta.benedetti@univaq.it (E.B.); margherita.alfonsetti@guest.univaq.it (M.A.); mquintiliani@unite.it (M.Q.); annamaria.cimini@univaq.it (A.C.); 2 Dompè Farmaceutici Spa, via Campo di Pile, 1, 67100 L’Aquila, Italy; laura.brandolini@dompe.com (L.B.); daniela.iaconis@dompe.com (D.I.); 3 Istituto di Ricerche Farmacologiche Mario Negri IRCCS, 20156 Milan, Italy; maddalena@marionegri.it; 4 Sbarro Institute for Cancer Research and Molecular Medicine, Department of Biology, Temple University, Philadelphia, PA 19122, USA

**Keywords:** nutraceutical, diet, brain, antioxidant, mitochondria

## Abstract

The mucolytic agent S-carboxymethylcysteine is widely used as an expectorant for the treatment of numerous respiratory disorders. The metabolic fate of S-carboxymethyl-L-cysteine is complex. Several clinical studies have demonstrated that the metabolism of this agent differs within the same individual, with sulfur oxygenated metabolites generated upon night-time administration. It has been indicated that this drug behaves like a free radical scavenger and that, in this regard, the sulfide is the active species with sulphoxide metabolites (already oxidized) being inactive. Consequently, a night-time consumption of the drug should be more effective upon daytime administration. Still, this diurnal variation in biotransformation (deactivation) is dependent on the genetic polymorphism on which relies the patient population capacities of S-carboxymethyl-L-cysteine sulphoxidation. It has been reported that those cohorts who are efficient sulfur oxidizers will generate inactive oxygenated metabolites. In contrast, those who have a relative deficiency in this mechanism will be subjected to the active sulfide for a more extended period. In this regard, it is noteworthy that 38–39% of Parkinson’s disease patients belong to the poor sulphoxide cohort, being exposed to higher levels of active sulfide, the active antioxidant metabolite of S-carboxymethyl-L-cysteine. Parkinson’s disease is a neurodegenerative disorder that affects predominately dopaminergic neurons. It has been demonstrated that oxidative stress and mitochondrial dysfunction play a crucial role in the degeneration of dopaminergic neurons. Based on this evidence, in this study, we evaluated the effects of S-carboxymethyl cysteine in an in vitro model of Parkinson’s disease in protecting against oxidative stress injury. The data obtained suggested that an S-carboxymethylcysteine-enriched diet could be beneficial during aging to protect neurons from oxidative imbalance and mitochondrial dysfunction, thus preventing the progression of neurodegenerative processes.

## 1. Introduction

The mucolytic agent S-carboxymethyl cysteine (SCMC) is widely used as an expectorant for the treatment of different respiratory diseases characterized by abnormal mucus secretion, including chronic obstructive pulmonary disease (COPD), a serious life-threatening pathology whose main feature is a persistent lung inflammation, where airway cells are subjected to chronic oxidative stress [1]. The bulk of clinical data highlights that SCMC is a well-tolerated treatment with a favorable safety profile that exerts its mucus-regulatory activity by promoting sputum clearance and by reducing the incidence of COPD exacerbations, thus improving patient’s quality of life [2].

Preclinical and clinical studies on mucus transport and mucus hypersecretions have demonstrated that SCMC was able to modify sputum rheology, thus normalizing mucus production and composition, increasing ciliary clearance, and improving histological aspects of broncho-tracheal mucosa [3,4,5]. Besides its mucus-regulatory activity, SCMC has been proven to possess a wide range of pharmacological properties both in humans and in animal models. The anti-inflammatory properties of the drug have been widely investigated both in vivo and in vitro, demonstrating that SCMC is effective in inhibiting airway injury in rats exposed to sulfur dioxide (SO2) [6,7] and in other pulmonary inflammation models that involve the activation of different cytokines including IL-8 and IL-6 [8,9]. More recently, it was reported that SCMC suppresses inflammation in human alveolar epithelial cells upon stimulation with TNF-α [10].

In vitro studies have reported a significant and dose-dependent reduction in adherence of *Moraxella catarrhalis* and *Streptococcus pneumoniae* to the human pharyngeal epithelial cells treated with SCMC [11,12], suggesting a role of the drug in inhibiting the attachment of bacteria to the upper respiratory tract. More recently, SCMC has been proven to modulate airways inflammation caused by rhinovirus [13], respiratory syncytial virus, and type A seasonal influenza virus [14] infections by decreasing the expression of intercellular adhesion molecule-1 (ICAM-1), which is the receptor for the major respiratory pathogens.

Increasing evidence shows that SCMC has relevant antioxidant properties. The in vitro SCMC-antioxidant activity has been evaluated in the free cellular system as well as in activated human polymorphonuclear neutrophils against some reactive oxygen species, all produced during lung inflammatory disorders; the data demonstrated a clear and selective scavenger activity [15,16].

SCMC is a non-natural sulfur-containing amino acid, formally a thioether derivative of cysteine. The lack of a free thiol group implies a different antioxidant mechanism of action as compared to cysteine itself or other commonly available mucolytic cysteine derivatives such as N-acetylcysteine (NAC). NAC contains one free sulfhydryl (thiol) group able to react with the glycoproteins disulfide bonds in mucus, thus increasing mucus viscosity; on the contrary, SCMC does not directly react with glycoproteins disulfide bonds but it can influence the mucus rheological properties by restoring the correct balance between sialo- and fuco-mucins (higher concentrations of the former and reducing the latter) through the intracellular stimulation of sialyltransferase activity [5,17]. It is assumed that NAC can protect cells against oxidative damage by directly scavenging via its free thiol group to form NAC disulfide, even if this narrative is not supported by clear scientific evidence [18]. NAC shares many characteristics of the parent amino acid, cysteine. Because of its redox instability, almost all extracellular cysteine is present in the oxidized cysteine status. Cysteine is the rate-limiting amino acid substrate for intracellular glutathione synthesis, and it has been shown that, at low concentrations, the primary antioxidant mechanism of thiol amino acids, like cysteine and NAC, is mediated by their ability to increase glutathione levels, rather than direct scavenging of reactive oxygen. On the other hand, SCMC like methionine, another sulphur-containing amino acid, has a thioether bond that can be oxidized by ROS to form the sulfoxide. Methionine is an efficient scavenger of almost all oxidizing species under physiological conditions, such as H202, hydroxyl radicals, peroxynitrite, chloramines, and hypochlorous acid. SCMC mechanism as a free radical scavenger was examined in the free cellular system as well as in activated human polymorphonuclear neutrophils [15]; data demonstrate that, similarly to methionine, the molecule is a potent and selective scavenger of OH- and HOCl, the most powerful free radicals in terms of tissue damage, since they can inactivate α1-antitrypsin by oxidizing the protein methionyl residues, which leads to an uncontrolled neutrophil elastase activity. The scavenger capacity of SCMC is related to the reactivity of its thioether group and is paralleled by the protection of a-1AT activity and the inhibition of IL-8 production, resulting in a decreased neutrophil-mediated inflammation [15].

Parkinson’s disease (PD) is the second most frequent neurodegenerative disorder, with prevalence increasing with age. The molecular mechanism of PD is still uncertain, but one relevant factor in the disease pathogenesis is oxidative stress [19,20]. Mitochondrial dysfunction and ROS accumulation occur in many neurodegenerative diseases including Alzheimer’s, Parkinson’s, and Huntington’s diseases. The crosstalk existing among oxidative stress and mitochondrial dysfunction plays a pivotal role in the onset and progression of neurodegenerative diseases [21].

Considering the well-established antioxidant properties of SCMC, in the present study we investigated its effects on an in vitro model of PD that we recently used to describe a new mechanism underlying the antioxidant activity of methionine [22]. Our previous study provided evidence that methionine could exert its antioxidant activity by stimulating the methionine sulfoxide reductase (Msr) system, an important cellular repair mechanism that protects against the deleterious effects of oxidative stress by reduction of oxidized methionine of both proteins and free amino acids. In this work, we explored the hypothesis that SCMC may share with methionine the property of activating the Msr system. For this reason, we investigated SCMC effects in a Parkinson’s disease in vitro model, where the pro-oxidant effect of 6-OHDA results in oxidative damage to proteins and mitochondria. In particular, we tried to dissect the underlying mechanisms to gain insights into the potential of SCMC treatment in slowing the progression of the disease by maintaining mitochondrial functionality.

## 2. Materials and Methods

### 2.1. Cellular Model

The human neuroblastoma cell line SH-SY5Y (ATCC, USA) was cultured in Dulbecco’s minimum essential medium (Corning, New York, NY, USA) supplemented with glutamine and antibiotics. Differentiated SH-SY5Y cells are broadly used for in vitro studies involving neuronal-like cells. Differentiated SH-SY5Y cells are low cost to culture, and the ethical concerns related to primary human neuronal culture are avoided. Moreover, since SH-SY5Y cells are human-derived, they present numerous human-specific proteins and isoforms that would not be intrinsically present in rodent primary cultures [23]. To induce the differentiation, SH-SY5Y were plated at 15,000 cells/cm^2^ and, after 24 h, were grown in 10 μM Retinoic acid of RA with 1% FBS for 3 days. After 3 days, the media was replaced with fresh 12-O-tetrade-canoyl-phorbol-13-acetate (TPA) (Sigma, St. Louis, MO, USA) for another 3 days of differentiation. For the setup of the in vitro PD model, the differentiated SH-SY5Y cell line was treated with 6-hydroxydopamine (6-OHDA) (Sigma) for 24 h after differentiation. SCMC and S-methyl-L-cysteine sulfoxide (SCMC-O) treatments were performed one hour before 6-OHDA stress and used at the final concentration of 0.25 mM while NAC was performed at the final concentration of 5 mM according to Yakamuro et al. [24].

### 2.2. Cell Viability

Cell viability was determined using Cell Titer One Solution Cell Proliferation Assay (Promega, Madison, WI, USA), a colorimetric method established on the quantity of formazan formed, as a function of viability. The plate was read at 492 nm using an ELISA plate reader, Spark (Tecan, Männedorf, Switzerland). Cells were seeded and treated as described in “Cellular model” paragraph. Cells have been treated with SCMC/SCMC-O/NAC for 1 h at concentration: 0.25 mM for SCMC/SCMC-O and 5 mM for NAC [25,26]. After these treatments, the cells were stressed with 6-OHDA at 35 µM concentration for 24 h. The assay was executed in quintuplicate. The results were expressed as absorbance.

### 2.3. Expression Analysis: RNA-Seq and Gene Clustering

Cells were cultured as described previously and RNA was extracted with Trizol (Invitrogen, Waltham, MA, USA) following the manufacturer’s instructions. Data were obtained from SH-SY5Y untreated cells as a control and cell treated either with 6-OHDA alone or in combination with SCMC and NAC. All the experiments were assayed in triplicate. RNA-seq experiments were performed at Lexogen, a biotech company expert in expression profiling technologies using the QuantSeq 3′ mRNA-Seq Library Prep Kit, which is a library preparation protocol designed to generate libraries of sequences close to the 3′ end of polyadenylated RNA. Data were aligned by the STAR tool (https://github.com/alexdobin/STAR/blob/master/doc/STARmanual.pdf), and differential expression analyses were performed with DESeq2 implemented in R (http://www.bioconductor.org/packages/release/bioc/vignettes/DESeq2/inst/doc/DESeq2.html#theory). Aggregated quality control and Trimming to remove various types of undesirable sequences (i.e., primers, poly-A tails) were executed with MULTIQC and Cutadapt tools separately (http://multiqc.info/; https://cutadapt.readthedocs.io/en/stable/). Clustering (Euclidean metrics) and functional analysis of differentially expressed genes was done by t-Mev (https://mev.tm4.org). The interactor’s network was built with STRING (http://string-db.org; [27]).

### 2.4. Western Blotting

Cells were seeded and treated as described in “Cellular model” paragraph. Cells were washed with cold 1× phosphate-buffered saline (PBS) buffer followed by detachment with scrapers. Pellets were collected and resuspended in cell lysis buffer containing protease and phosphatase inhibitor. After, the lysate was collected and maintained on ice for 30 min. Finally, total protein was extracted by centrifugation at 14,000 RPM for 30 min at 4 °C. Protein amount was measured using BCA kit as previously reported [28] and gel electrophoresis was executed (loading 30 µg/lane or 50 µg/lane for 4-HNE) on 8–15% polyacrylamide denaturing gels. Proteins were transferred onto polyvinylidene difluoride membranes and then blocked in blocking solution (Biorad, Hercules, CA, USA) for 5 min at RT. The membranes were then incubated with: anti-p-TrkB (1:1000), anti-TrkB (1:500), anti-BDNF (1:1000), anti-p-CREB (1:1000), anti-CREB (1:1000), anti-p-AKT (1:1000), anti-AKT (1:500), anti-p-Nrf2 (1:4000), anti-Nrf2 (1:1000), anti-4-HNE (1:1000), anti-Sirt1 (1:500), anti-p-Foxo3A (1:1000), anti-Foxo3A (1:1000), anti-Msrb2 (1:500), anti-Opa1 (1:500), anti-Mfn1/2 (1:1000), anti-Drp1 (1:500) at 4 °C overnight and after three washes in TBS-T, further incubated with 1:20,000 horseradish peroxidase-conjugated anti-rabbit IgG or anti-mouse IgG. The protein bands were visualized using chemiluminescence reagent according to the manufacturer’s instructions. Alliance Uvitec was used to image chemiluminescent bands, and to perform the analysis of each band intensity, NIH ImageJ program was used. Anti-β-actin HRP-conjugate (1:10,000) or anti-GAPDH (1:4000) were used as a loading control, and the relative peak intensity was normalized with respect to the loading control. For the phosphorilated forms, the total forms were used to normalize.

### 2.5. OxyBlot

Cells were seeded and treated as described in “Cellular model” paragraph. Protein carbonyls were assayed by WB analyses following the manufacturer’s protocol (OxyBlot Protein Oxidation Detection Kit). An amount of 30 μg of protein lysate was mixed with 2,4 dinitrophenylhydrazine and blotted using a primary antibody specific to dinitrophenylhydrazone-derivatized residues (OxyBlot 1:150) and a secondary antibody against the first one (OxyBlot 1:300). Protein carbonyls were visualized by Alliance Q9 MINI WL and revealed using enhanced chemiluminescence and quantified by densitometry using software ImageJ. The bands obtained were normalized on Coomassie blue staining of the total gel.

### 2.6. Mitotracker Deep Red and Quantification of Mitochondrial Fragmentation

MitoTracker is a fluorescent dye that stains mitochondria in live cells, and its accumulation is dependent upon membrane potential. In particular, cells were seeded on the coverslips at a density of 1.5 × 10^4^. After treatments, the cells were washed three times with PBS. Then, the cells were incubated with Mitotracker deep Red (Invitrogen, USA) in HBSS buffer at a concentration of 1 µM for 15 min. Then, cells on coverslips were washed three times in PBS and then fixed with 3.7% formaldehyde in PBS for 15 min at RT. After further washes in PBS, coverslips were mounted with Vectashield mounting medium containing DAPI nuclear dye. The observation was performed with a confocal microscope Leica TCS SP5, and images were acquired with LCS Leica confocal software SP5 (Leica microsystem, Germany). Quantitative determination of mitochondrial fragmentation was performed as previously described [29]. Mitochondria shorter than 2 μm were considered as fragmented and those longer than 5 μm as filamental mitochondria. The mitochondrial images were binarized by the threshold module using ImageJ, and these binary images were converted to images 1 pixel wide by the skeletonize module. Regarding mitochondrial length, it was calculated utilizing the analyze particles module. All the analyses were performed by an investigator blind to the experiment on at least 25 randomly chosen cells [30].

### 2.7. Mitosox

Cells were stained with Mitosox Red reagent, which is a novel fluorogenic dye specifically targeted to mitochondria in live cells. Mitosox Red reagent permeates live cells where it selectively targets mitochondria. It is rapidly oxidized by superoxide but not by other reactive oxygen species (ROS) and reactive nitrogen species (RNS). When the red reagent is oxidized by superoxide, it exhibits red fluorescence. Cells were marked with Mitotracker Green FM (200 nM) and washed three times with PBS and then incubated with MitoSox (Invitrogen, USA) in HBSS buffer at 1 µM concentration for 10 min. After different washes in PBS, coverslips were mounted with Vectashield mounting medium containing DAPI nuclear dye. The observation was performed with a confocal microscope Leica TCS SP5, and images were acquired with LCS Leica confocal software SP5 (Leica microsystem, Wetzlar, Germany). The data analyses were performed using NHI ImageJ and the fluorescence intensity for Mitotracker green was normalized on the nuclei (DAPI), while the fluorescence intensity for Mitosox Red was normalized on the normalized Mitotracker intensity.

### 2.8. TMRM

Healthy mitochondrial membranes maintain a difference in electrical potential between the interior and exterior of the organelle, referred to as a membrane potential. Tetramethylrhodamine, methyl ester (TMRM) is a cell-permeant dye that accumulates in active mitochondria with intact membrane potentials. If the cells are healthy and have functioning mitochondria, the signal is bright. Upon loss of the mitochondrial membrane potential, TMRM accumulation ends and the signal fades or disappears. TMRM assay was performed by IncuCyte Live System. The cells were seeded on the multiwell plate at a density of 2 × 10^4^ cells for well (optimized to have a better staining). After treatment, the cells were incubated with TMRM reagent (200 nM, Invitrogen, USA) in PBS for 15 min at 37 °C. After incubation, the multiwell was transferred to the IncuCyte controller for 24 h.

### 2.9. Seahorse Assay

The Seahorse XF96e Extracellular Flux Analyzer (Agilent Technologies, CA, USA) was used to generate the bioenergetic profiles of differentiated neuroblastoma SH-SY5Y cell lines upon different treatments. Live-cell analyses of oxygen consumption rate (OCR) and extracellular acidification rate (ECAR) were measured using the Mito Stress test (Agilent, USA). Cells were cultured on a Seahorse XF96 cell culture plate at a density of 5.0 × 10^4^ cells/well (cell density was optimized to ensure a proportional response of FCCP with cell number) and grown overnight in DMEM 10% of FBS, then differentiated as described above. After complete differentiation, cells were treated as described above. On the day before the Seahorse assay, the cartridge was hydrated and incubated overnight at 37 °C in the absence of CO_2_. On the day of the assay, cell medium was replaced with freshly prepared unbuffered DMEM pH 7.4 (XF Assay Medium; Agilent Technologies, USA) supplemented with 5 mM glucose and 1 mM sodium pyruvate (Agilent Technologies, USA), and incubated for 1 h at 37 °C without CO_2_. After four baseline measurements for the oxygen consumption ratio, cells were sequentially challenged with injections of Mito Stress drugs prepared following the manufacturer’s instructions. The final concentrations used for each drug were 1 μM oligomycin (ATP synthase inhibitor), 1 μM FCCP (mitochondrial respiration uncoupler), and 0.5 μM rotenone/antimycin (complex I and III inhibitors). For the normalization in port D, Hoechst 33342 solution was injected, and at the end of the run, the plate was read using a microplate reader (Infinite Tecan, USA). The data and graphs generated at the end of the Mito Stress assay were extracted using Wave software.

### 2.10. Statistical Analysis

Data are mean ± SD of three or five different experiments. Statistical analysis was performed by one-way ANOVA following Tukey’s post-hoc. While for grouped analyses (TMRM), two-way ANOVA was used following by Tukey’s post-hoc. The level of significance was set at *p* < 0.05.

## 3. Results

In the first series of experiments, to setup the PD in vitro model, the 6-OHDA dose-response curve was performed (Figure S1). Cell viability dose-response curves for SCMC and the oxidized form SCMC-O in combination with 6-OHDA (35 µM) were performed (Figure S2). Based on these results, SCMC and SCMC-O were used at 0.25 mM.

PD is a progressive neurodegenerative disease characterized by mitochondrial dysfunction and loss of dopaminergic neurons mainly due to the accumulation of reactive oxygen species (ROS). Since SCMC, a free radical scavenger, is slowly metabolized by Parkinsonian neurons, we decided to deeper investigate the role of this protein in an in vitro model of PD.

**Figure 1:**
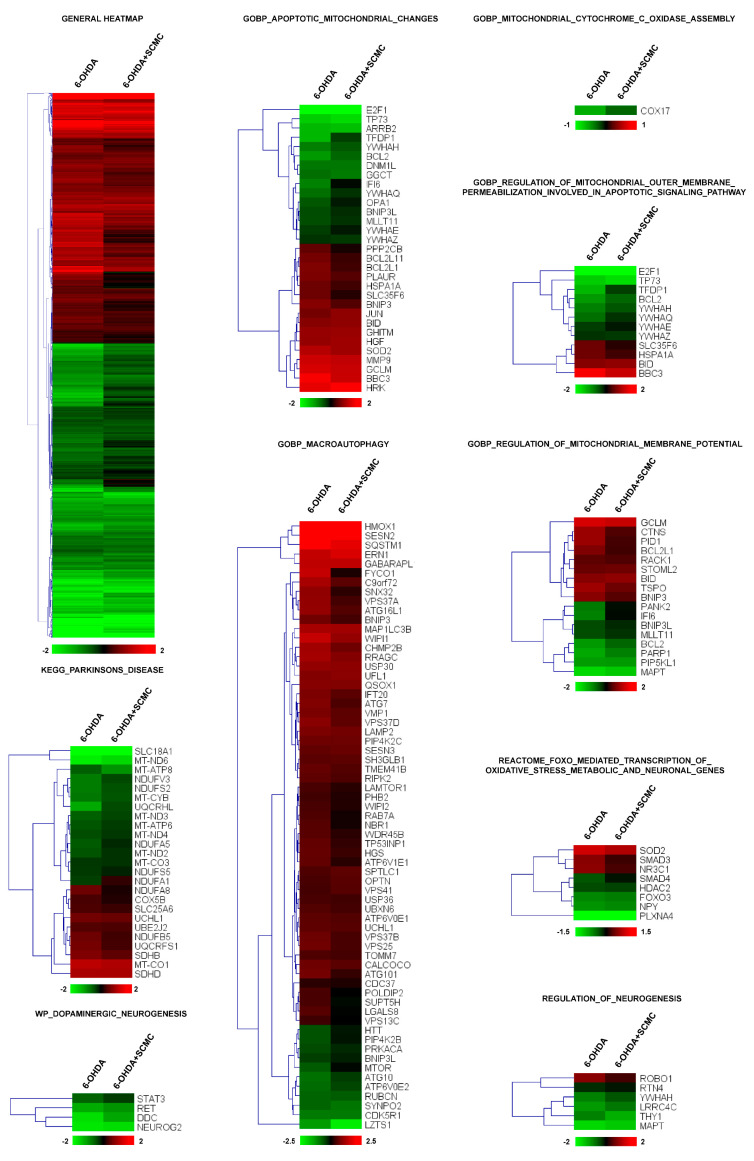
Heatmap of hierarchical clustering of the selected pathways. All the genes of the pathway that are significantly affected by 6-OHDA (adjusted *p* < 0.05) are reported in the heatmaps. Color scale represents log2 ratios of the expression levels in the indicated condition versus control. Color scale limits are indicated in the lower box.

To this aim, we performed RNA-sequencing experiments on differentiated SH-SY5Y cells treated with 6-OHDA plus SCMC and compared the expression profile to that obtained from cells treated with 6-OHDA alone or SCMC-O. More than 3000 transcripts were dysregulated by 6-OHDA (adjusted *p* < 0.05) and, interestingly, cluster analysis revealed that the general pattern of gene regulation was reverted by SCMC treatment (general heatmap in Figure 1). To analyze deeper the biological processes modulated by the treatments, a functional analysis of differentially expressed genes was performed by t-Mev and, interestingly, we found in GO a significant number of genes involved in mitochondrial functions, oxidative stress, apoptosis, macroautophagy, PD, and neurogenesis. Macroautophagy is a molecular process associated with PD. Sixty-five transcripts were modulated by SCMC, suggesting a novel role of this molecule in the pathway.

More than 60 transcripts dysregulated by 6-OHDA and recovered by SCMC are associated with mitochondrial dynamics and apoptosis, among these genes, *Bcl2*, *Cox17*, *Ifi6*, and *Opa1* were downregulated by 6-OHDA and were recovered by SCMC treatment. The same occurs for several transcripts upregulated by 6-OHDA such as *Sod2*, *Hspa1a*, and *Tspo* (Figure 1 and Figure S3).

Oxidative stress was also dysregulated by 6-OHDA and interestingly we found a group of FoxO-associated transcripts that were recovered by SCMC (Figure 1, Reactome_FOXO_mediated_transcription_of_oxidative_stress_metabolic_and_neuronal_genes). Of great interest was the presence of a functional group associated with Parkinson’s disease and neurogenesis, in which related transcripts were almost all recovered by SCMC (Figure 1, KEGG_Parkinsons_disease).

Altogether, these results suggested that SCMC is strictly involved in the key molecular pathways associated with neuronal cell behavior observed in Parkinson’s disease.

**Figure 2:**
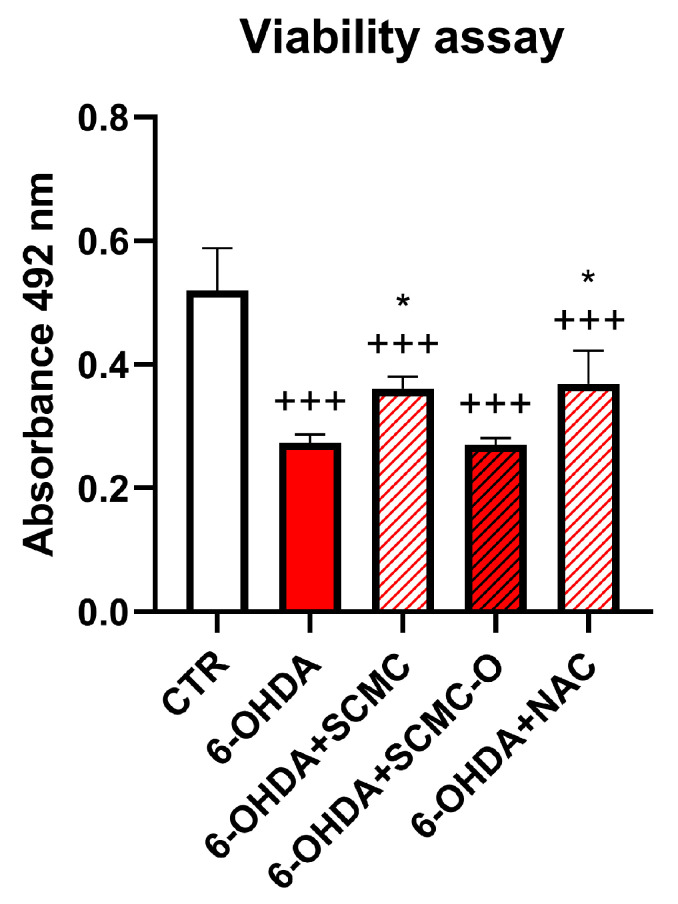
Cell viability assay for differentiated SH-SY5Y upon 6-OHDA, SCMC, NAC, and SCMC-O. Data are mean ± SD of three different experiments. *: *p* < 0.05 vs. 6-OHDA; +++: *p* < 0.0001.

In light of the RNAseq results, we decided to compare the effect of SCMC with a well-known antioxidant, NAC. In Figure 2, a cell viability assay for 6-OHDA, SCMC, SCMC-O, and NAC treatments on differentiated SH-SY5Y is reported. It is possible to observe that 6-OHDA exerted a strong cytotoxic activity, while SCMC as NAC efficiently counteracted this effect; in contrast, SCMC-O was not effective.

**Figure 3:**
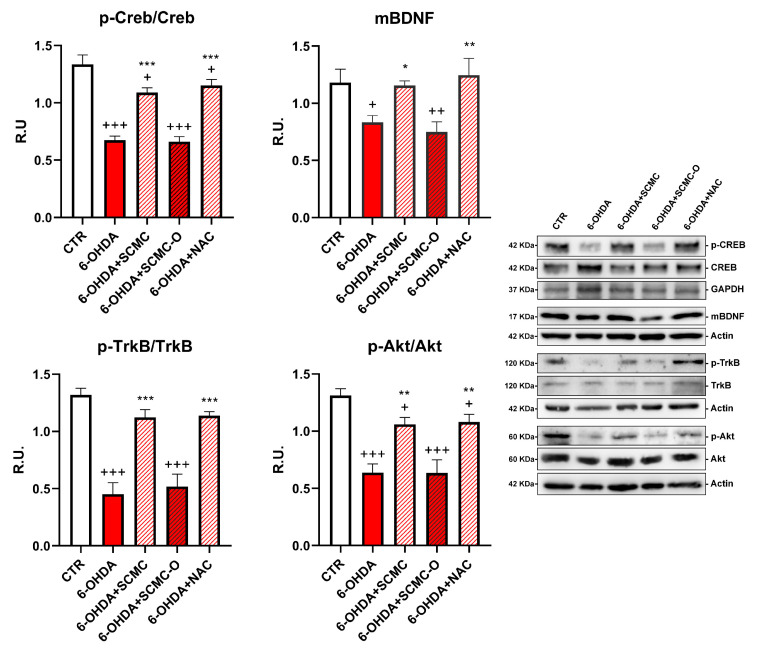
WB analyses for the neuroprotective pathways p-Creb, mBDNF, p-AKT, and p-TrkB. Data are mean ± SE of three different experiments; *: *p* < 0.05; **: *p* < 0.005, ***: *p* < 0.0001 vs. 6-OHDA; +: *p* < 0.05; ++: *p* < 0.005, +++: *p* < 0.0001 vs. CTR.

Neuronal survival is triggered by neurotrophins supply and signaling, including brain-derived neurotrophic factor (BDNF). Upon 6-OHDA, a significant decrease of the mature form of BDNF (mBDNF) was observed. SCMC was able to counteract this effect as NAC, while the oxidized form SCMC-O was not effective. In parallel, p-TrkB, p-CREB, and p-AKT (Figure 3) sharply decreased by 6-OHDA treatment while the treatments with SCMC or NAC restored the control levels for the analyzed proteins. The SCMC-O was ineffective in counteracting 6-OHDA effects.

**Figure 4:**
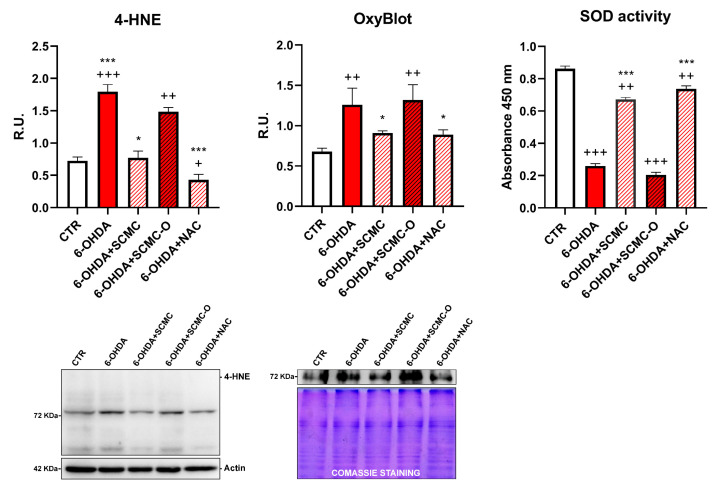
4-HNE, OxyBlot assay, and SOD activity analyses are shown. Data are mean ± SD of three different experiments. *: *p* < 0.05; ***: *p* < 0.0001 vs. 6-OHDA; +: *p* < 0.05; ++: *p* < 0.005; +++: *p* < 0.0001 vs. CTR. A representative figure for 4-HNE and Oxyblot are reported.

**Figure 5:**
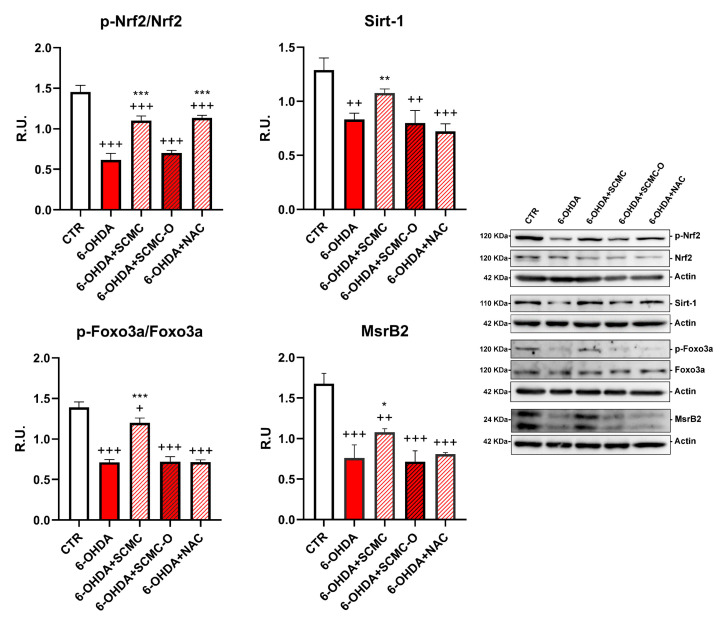
WB analyses p-Nrf2 and Sirt-1/Foxo3a/MsrB2 pathway. A representative figure is shown. Data are mean ± SD of three different experiments. *: *p* < 0.05; **: *p* < 0.005; ***: *p* < 0.0001; *p* < 0.005 vs. 6-OHDA; +: *p* < 0.05; ++: *p* < 0.005; +++: *p* < 0.0001 vs. CTR.

It is recognized that 6-OHDA increases oxidative stress markers as well as oxidative damage. In this regard, in Figure 4, the Oxyblot analysis for oxidized proteins and HNE protein adducts are reported together with superoxide dismutase (SOD) activity. 6-OHDA strongly increased oxidized proteins and HNE protein adducts while it strongly decreased SOD activity. Notably, SCMC and NAC both counteracted these effects, while SCMC-O was not able to. In Figure 5, the Western blot analysis for the total and phosphorylated forms of Nrf2, Sirt-1, FOXO3a, as well as the downstresm effector MSrB2 mitochondrial enzyme were assessed. 6-OHDA decreased all the players of this antioxidant pathway, SCMC or NAC almost restored the control values, while again SCMC-O was not able to.

**Figure 6:**
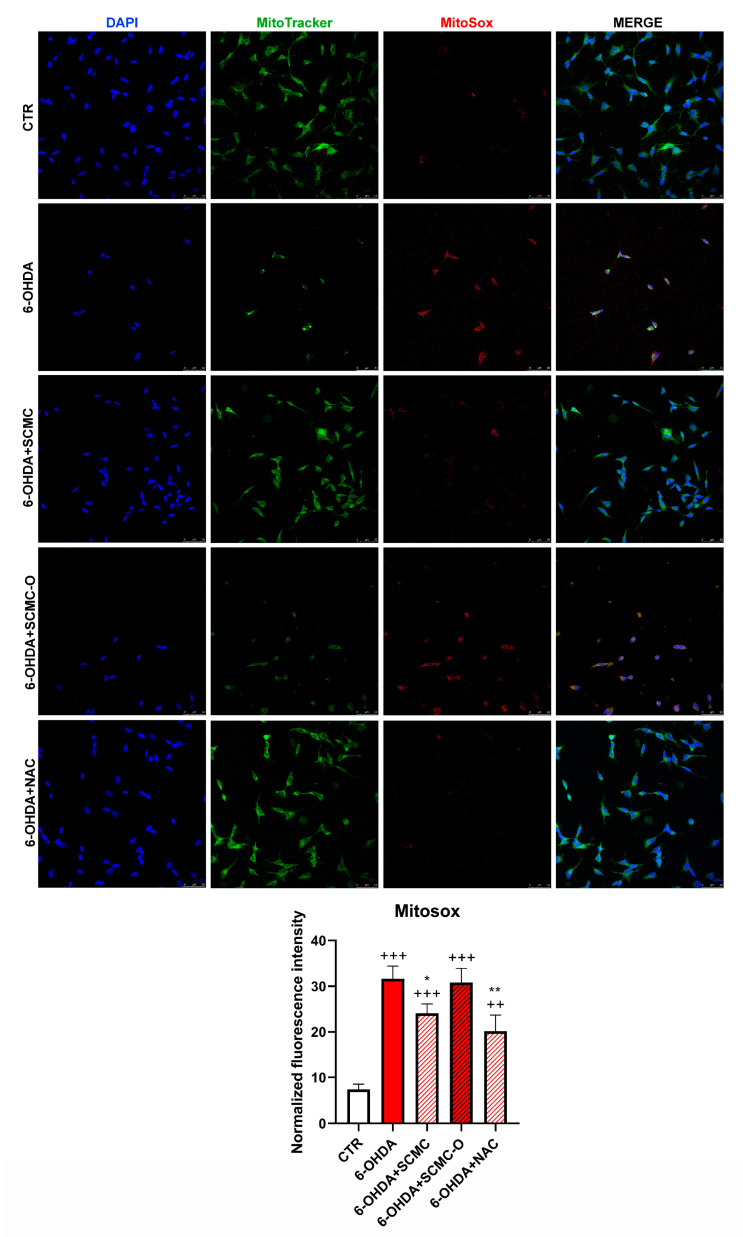
Mitotracker green with Mitosox. The graph shows the normalized fluorescence intensity MitoSox/Mitotracker normalized on nuclei. Data are mean ± SD of three different experiments. *: *p* < 0.05; **: *p* < 0.005 vs. 6-OHDA; ++: *p* < 0.005; +++: *p* < 0.0001 vs. CTR. Scale Bar: 50 µm.

The increase of oxidative stress is generally accompanied by mitochondrial damage; therefore, mitochondrial functionality was analyzed. In Figure 6, mitochondrial number and mitochondrial oxidative species were evaluated by Mitotracker/MitoSox using confocal laser microscopy. 6-OHDA strongly decreased the mitochondrial number and increased MitoSox. The presence of SCMC or NAC restored these parameters, while SCMC-O was ineffective.

**Figure 7:**
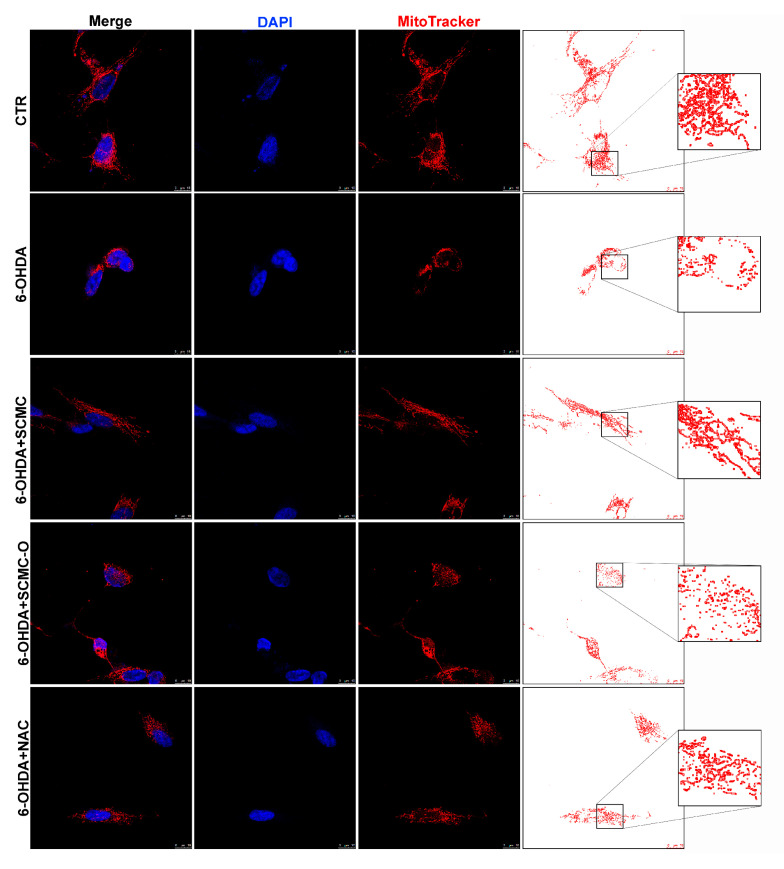

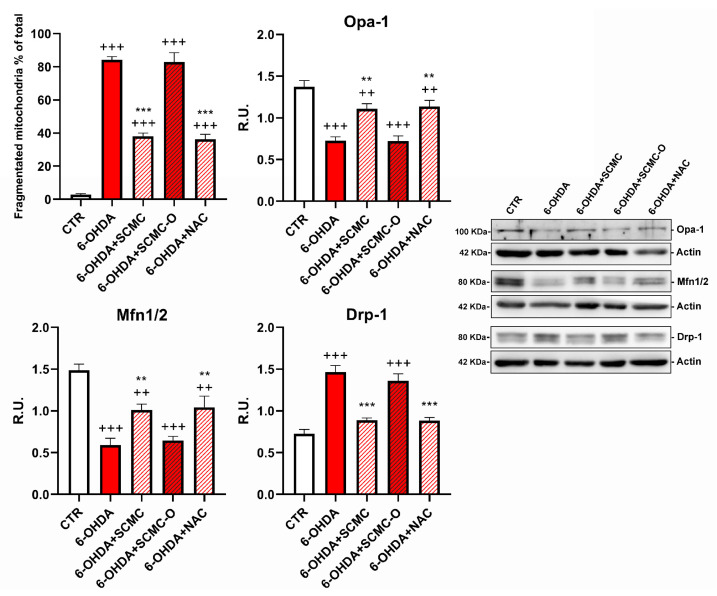
DAPI, Mitotracker deep red. The masks generated by ImageJ representative images are reported. In the red circle, the zoom in is reported to better appreciate the mitochondrial morphology. WB analyses for fission and fusion pathways. A representative WB figure is shown. Data are mean ± SD of three different experiments. *** *p* < 0.0001, ** *p* < 0.005 vs. 6-OHDA; +++ *p* < 0.0001; ++ *p* < 0.005 vs. CTR.

Mitotracker deep Red and Western blotting analysis for fusion/fission proteins were used to evaluate mitochondrial morphology and dynamics, which are essential for main-taining mitochondrial function. Cells treated with 6-OHDA or 6-OHDA and SCMC-O showed shorter and smaller size, suggesting mitochondrial fragmentation. SCMC and NAC were able to partially inhibit the mitochondrial fragmentation induced by 6-OHDA, maintaining the mitochondrial morphology (Figure 7). Mfn-1/2 and Opa-1 have a pivotal role in the control of mitochondrial fusion, while Drp-1 plays a key role in mitochondrial fission. In fact, mitochondrial morphology is likely to be the result of the interplay between mitochondrial fission and fusion. Upon 6-OHDA stress alone or in combination with SCMC-O, fusion proteins (Mfn-1/2 and Opa-1) resulted as decresead while the fission protein (Drp-1) was increased. SCMC and NAC partially counteracted the fission/fusion process altered by 6-OHDA (Figure 7).

**Figure 8:**
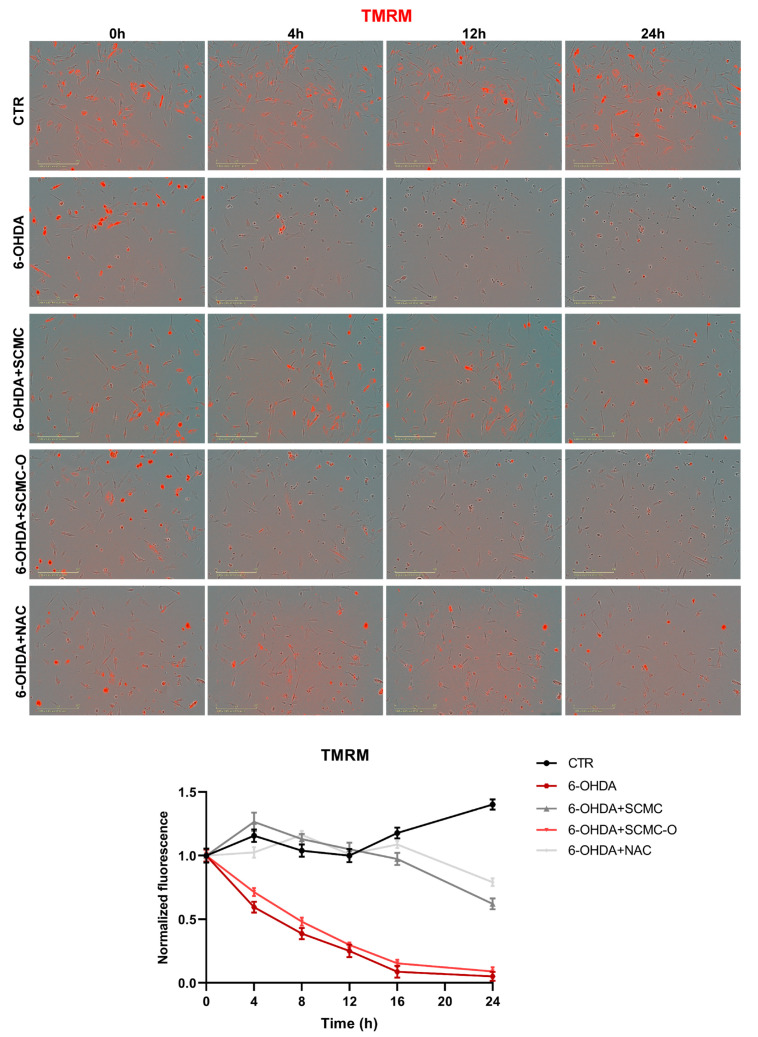
TMRM in live-cell by Incucyte system at 0 h, 4 h, 12 h, and 24 h time points. Representative images and relative normalized fluorescence graph are shown. Data are mean of three different experiments ± SD. The significance is reported in Table S1. Scale bar: 200 µm.

In Figure 8, mitochondrial membrane potential (MMP) was evaluated by TMRM live-imaging assay. 6-OHDA significantly decreased MMP, while SCMC and NAC were able to restore this parameter to control the condition, while in SCMC-O this effect was not observed. It is worth noting that MMP starts to decrease already at 4 h of 6-OHDA treatment, and SCMC shows its protective effects in maintaining MMP as early as 4 h after the 6-OHDA insult. In parallel, Seahorse experiments, assessing mitochondrial respiration, were performed.

**Figure 9:**
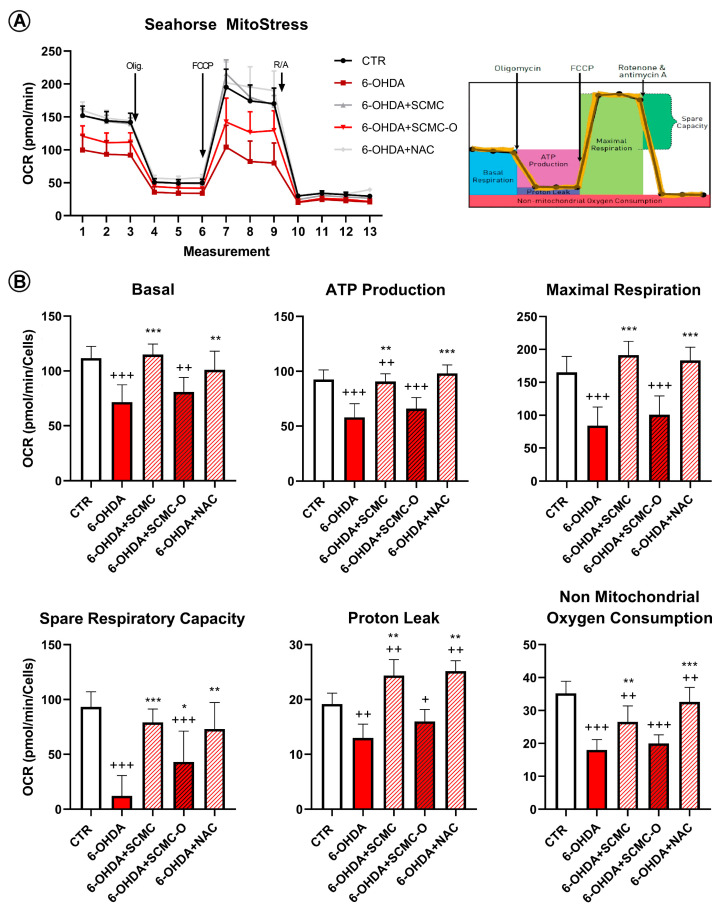
Mitochondrial bioenergetic profile in CTR and treated cells. (**A**) Seahorse XF Cell Mito Stress profile illustrated the key parameters of mitochondrial function upon the injection of different drugs. (**B**) Graph relative to basal respiration, ATP production, maximal respiration, and non-mitochondrial respiration in CTR and treated cells. Data are mean± SD of three different experiments. *** *p* < 0.0001, ** *p* < 0.005, * *p* < 0.05 vs. 6-OHDA; +++ *p* < 0.0001; ++ *p* < 0.005, + *p* < 0.05 vs. CTR.

Mitochondrial functionality in our cell model using Mito Stress assay by Seahorse Extracellular Flux Analyzer was evaluated to characterize the cell bioenergetics profile upon the different treatments (Figure 9). In particular, in Figure 9A, the time course and live measurement of OCR with injections of stressor compounds is shown. OCR resulted as strongly reduced by 6-OHDA, whereas the co-presence of SCMC was able to counteract this effect in contrast with SCMC-O (Figure 9A).

With the injection of oligomycin, cells treated with 6-OHDA and SCMC as well as NAC maintained much higher oxygen consumption than 6-OHDA alone or SMC-O, leading to significant differences in ATP production through mitochondrial respiration (Figure 9B). The reduction in proton leak induced by 6-OHDA was reverted by all the treatments tested. The induction of maximal respiration with FCCP injection resulted in decreased oxygen consumption upon 6-OHDA, while the combined treatment showed a behavior similar to CTR condition (Figure 9B).

## 4. Discussion

The metabolism of SCMC, an extensively used and widely available mucoactive drug, is complex. Clinical studies demonstrated that the metabolism of the drug differs within the same individual, with sulfur-oxygenated metabolites being generated upon night-time intake [31]. The sulfide is the active product with the sulphoxide metabolites (already oxidized) being inactive. It has been shown that a night-time consumption of the drug is more efficient compared to daytime administration. Still, this diurnal deactivation is dependent on an essential genetic polymorphism with a patient population with a spread of S-carboxymethyl-L-cysteine sulphoxidation capacities [32].

In this work, we dissected the molecular pathways underlying the antioxidant effects of SCMC, in a cellular model characterized by high levels of oxidative stress and cell death that can be considered an in vitro model of PD. We demonstrated that SCMC can act as a strong antioxidant with an efficiency comparable to NAC in protecting differentiated SH-SY5Y against oxidative stress (6-OHDA challenge). The RNAseq analyses indicate that different pathways appear modulated by 6-OHDA and recovered by SCMC; in particular, pathways involved in apoptosis promotion and oxidative stress appeared up-regulated by the neurotoxin and restored by SCMC. It is worth noting the behavior of SOD2, whose transcript appears upregulated by 6-OHDA and partially recovered by SCMC. This finding agrees with a network analysis identifying the increase of SOD2 mRNA as a potential biomarker for PD [33]. However, the analyses of the enzymatic activity of SOD2 showed a significant decrease, recovered by SCMC treatment, supporting the evidence of the enzyme sensitivity and inducibility under oxidative stress conditions.

PD is a disabling progressive disease with a strong impact on the patients’ quality of life. To date, there are no definitive therapies, but only symptomatic therapies that do not slow down the disease progress, which is mainly due to the progressive loss of dopaminergic neurons primary for the increase of oxidative stress leading to cellular dysfunction and neuroinflammation.

Several nutraceutical compounds have been proposed as an adjuvant treatment to ameliorate the oxidative stress component of the disease [34,35,36,37,38], however, the effect of an antioxidant is not long-lasting, and therefore reiterated administrations are needed. SCMC is rapidly inactivated in inactive oxygenated metabolites by efficient sulfur oxidizers. In PD patients, SCMC is poorly metabolized, thus functioning for longer times [39].

Recent studies from animals and cellular PD models indicated the participation of proteins linked to autosomal dominant PD, particularly α-synuclein and LRRK2, in the autophagy pathway [40,41]. Also, proteins related to recessive PD, such as PINK1 and PARKIN, have been implicated in the process of mitophagy. Autophagy can be highly specific, and in PD a specific autophagy-targeting mitochondria has also been reported [42,43]. Notably, it appears that SCMC was able to recover, in RNAseq analisis and GO pathways, the autophagy pathway, as well as mitochondrial functionality.

In agreement, all the biochemical data obtained point towards a direct antioxidant activity by increasing pro-survival pathways, such as BDNF signaling, and decreasing oxidative stress and protein oxidation.

Methionine sulfoxide reductases are key mitochondrial-localized endogenous antioxidative enzymes that can scavenge oxidizing species by catalyzing the methionine (Met)-centered redox cycle (MCRC) [44]. In this work, we focused our attention on the less studied MrsB2 mitochondrial isoform, demonstrating that SCMC can reduce mitochondrial ROS level through the SIRT1/pFOXO3a/sirtuin/MsrB2 pathway. MsrB2 has a protective role against oxidative stress and mitochondrial homeostasis, playing a crucial role in the antioxidant response by repairing methionine-oxidized proteins and catalyzing the methionine oxidation/reduction cycle [45,46,47]. MsrB2 levels decrease with age and in neurodegenerative pathological conditions, suggesting that a decline in the activity of this enzyme contributes to increased oxidative stress. SCMC, like methionine (antioxidants-1340554), the principal substrate of MsrB2, shares the thiother functional group, while this did not happen with NAC, which does not use the Sirt-1/Foxo3a/MsrB2 pathway for protecting cells but uses the activation of Nrf2 (as observed in WB analyses) [48]. The transcription factor Nrf2 binds to the antioxidant responsive element (ARE) and the activation of this pathway defends cells from oxidative stress-induced cell death [49]. Besides the typical initiation of detoxification enzymes, Nrf2-ARE induction leads to higher cellular energetics and redox potential, inhibitory neurotransmitter signaling, and metabolic processes.

It is worth noting that the progression of neurodegenerative disorders, including PD, is due to ROS accumulation, which leads to neuronal death. For this reason, lowering the ROS may result in a slower progression of the disease and, consequently, longer effects of the conventional therapies. It has been suggested that mitochondrial superoxide overexpression can be responsible for the neurotoxicity related to neurodegenerative processes. Mitochondria are believed to be a main source of ROS from aerobic respiration under physiological and many pathophysiological disorders. In agreement, herein, we provide evidence that SCMC is as potent as NAC in protecting mitochondria against 6-OHDA injury by preventing mitochondria fragmentation and lowering mitochondrial oxygen species (Mitosox). Furthermore, SCMC and NAC inhibited the 6-OHDA-induced oxidative stress through the induction of mitochondrial fusion proteins (Mfn1/2 and Opa-1) and the inhibition of fission protein (Drp-1). In agreement with these results, SCMC behavior on the bioenergetic profile resulted in being comparable to NAC behavior in counteracting the reduction of OCR induced by 6-OHDA, as reported in Seahorse assay.

Moreover, SCMC by activating neuroprotective pathways (p-CREB, mBDNF, p-TRKb) was able to rescue cells from 6-OHDA-induced cell death. In line with the proposed antioxidant mechanisms, both SCMC and NAC showed the ability to modulate Nrf2 signaling and SOD, while decreasing oxidized proteins under 6-OHDA insult. Moreover, upon 6-OHDA, mitochondrial impairment (as highlighted by Seahorse analyses, TMRM, Mitotracker), probably related to the oxidative condition (increased MitoSox and oxidized protein assayed by Oxyblot), is apparent, concurring together with neurotrophins deficit in dopaminergic neurons. All these effects were counteracted by SCMC, leading to neuronal survival.

In mammals, Msr enzymes are ubiquitously expressed even though their role is not yet fully characterized [45]. The direct antioxidant effect of SCMC, together with its ability to stimulate the protective Msr pathway, suggests a potential use of SCMC in all conditions characterized by oxidative stress and mitochondrial dysfunction, such as neurodegenerative disorders, COPD, and lung inflammatory diseases for the recovery of mitochondrial functionality and for counteracting oxidative stress. Basing on the results obtained, we can postulate that SCMC could represent a potential preventive treatment for PD, i.e., as a dietary supplement. Further studies will be focused on exploring the in vivo pharmacological properties of SCMC in neurological disorders.

## Data Availability

The data that support the findings of this study are available from the corresponding author, upon reasonable request.

## Supplementary Materials

The following are available online at https://www.mdpi.com/article/10.3390/biomedicines9101467/s1, Figure S1: Dose response curve for 6-OHDA at different concentrations. ++ *p* < 0.005; +++ *p* < 0.0001 vs. CTR, Figure S2: Dose response curve for SCMC and SCMC-O at different doses. * *p* < 0.04 vs. 6-OHDA; ++ *p* < 0.005; +++ *p* < 0.0001 vs. CTR, Figure S3: Heatmap of hierarchical clustering of the selected pathways. Color scale represents log2 ratios of the expression levels in the indicated conditions vs. CTR. Color scale limits are indicated in the boxes below the respective heatmap, Table S1: Significance data relative to TMRM analyses (Figure 8) at different time points.
