# Supplementary material for: S-Carboxymethyl Cysteine Protects against Oxidative Stress and Mitochondrial Impairment in a Parkinson’s Disease In Vitro Model
Source: Biomedicines. 2021 Oct 14;9(10):1467. doi: 10.3390/biomedicines9101467 (PMC8533464; doi:10.3390/biomedicines9101467)
Supplement: Supplementary file 1 [file biomedicines-09-01467-s001.zip › biomedicines-1411791-supplementary.pdf]

Supplementary Figures

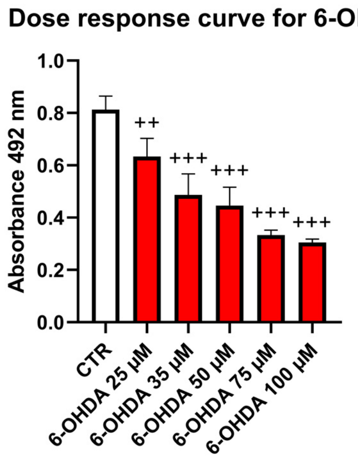

Figure S1. Dose response curve for 6-OHDA at different concentrations. ++p<0.005; +++p<0.0001 vs CTR.

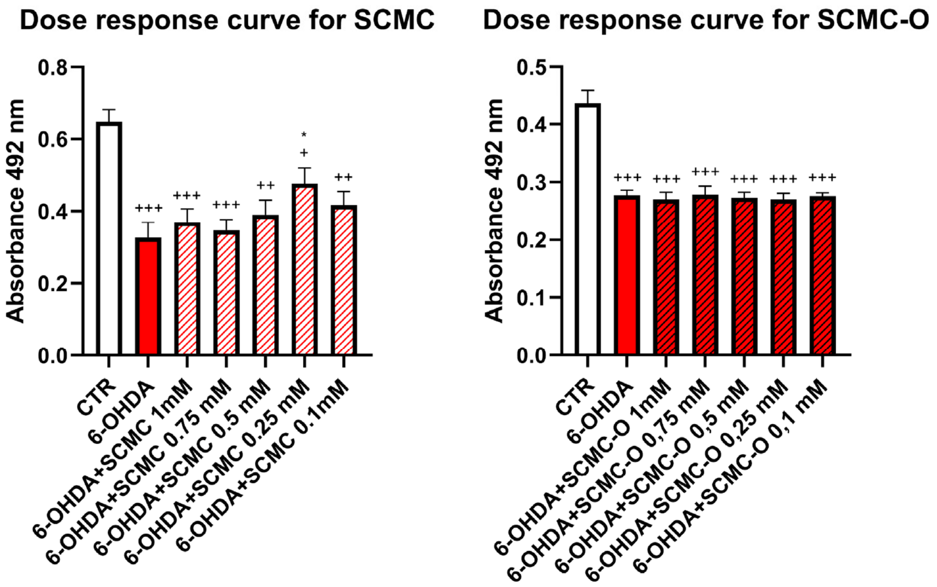

Figure S2. Dose response curve for SCMC and SCMC-O at different doses. *p<0.04 vs 6-OHDA; ++p<0.005; +++p<0.0001 vs CTR.

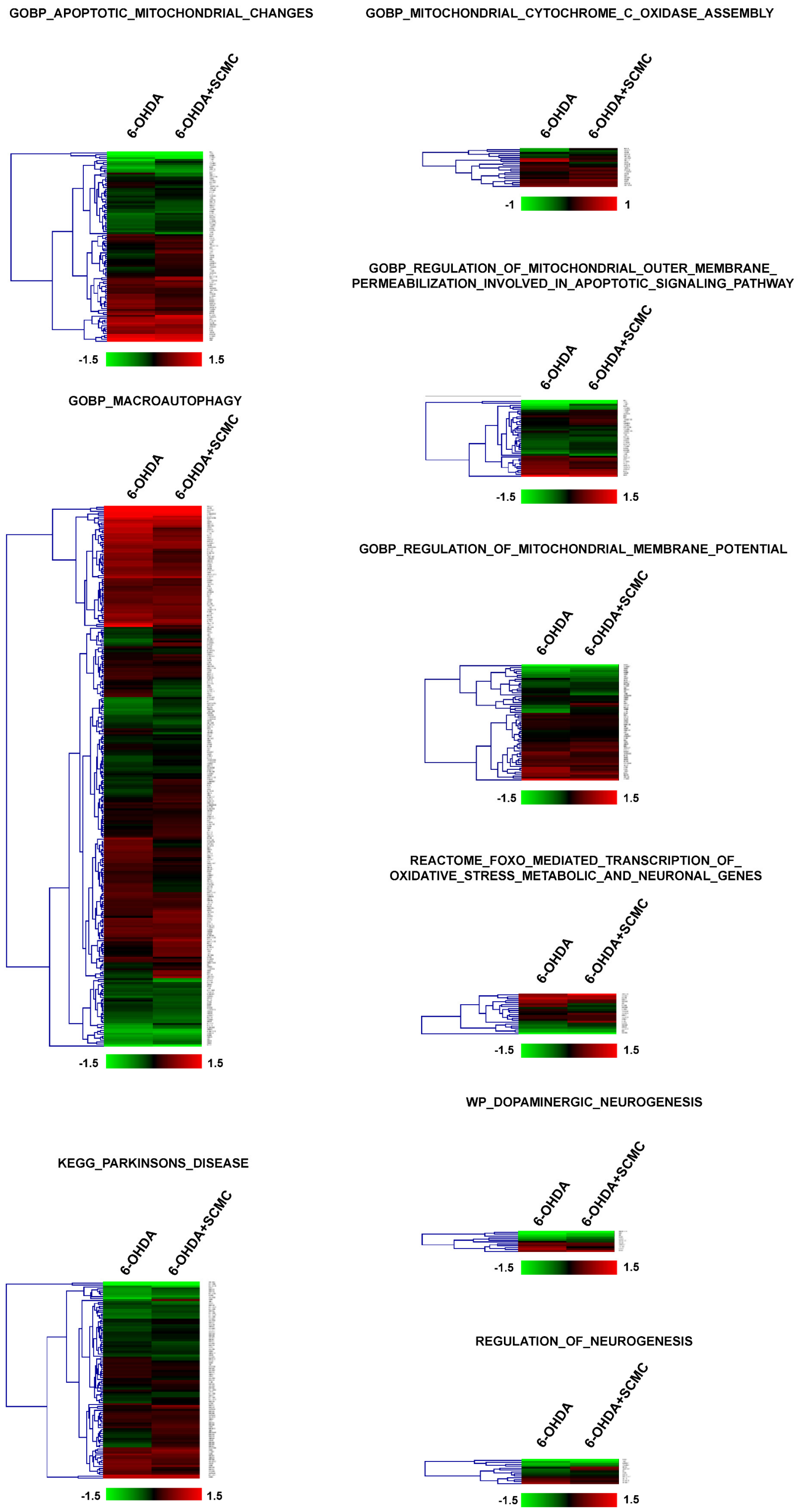

Figure S3: Heatmap of hierarchical clustering of the selected pathways. Color scale represents log2 ratios of the expression levels in the indicated conditions vs CTR. Color scale limits are indicated in the boxes below the respective heatmap.

0						0					
CTR vs. 6-OHDA	0	-0,1506 to 0,1506	No	ns	>0,9999						
CTR vs. 6-OHDA+SCMC	0	-0,1506 to 0,1506	No	ns	>0,9999	6-OHDA vs. 6-OHDA+SCMC	0	-0,1506 to 0,1506	No	ns	>0,9999
CTR vs. 6-OHDA+SCMC-O	0	-0,1506 to 0,1506	No	ns	>0,9999	6-OHDA vs. 6-OHDA+SCMC-O	0	-0,1506 to 0,1506	No	ns	>0,9999
CTR vs. 6-OHDA+NAC	0	-0,1506 to 0,1506	No	ns	>0,9999	6-OHDA vs. 6-OHDA+NAC	0	-0,1506 to 0,1506	No	ns	>0,9999
4						4					
CTR vs. 6-OHDA	0,5608	0,4102 to 0,7114	Yes	****	<0,0001						
CTR vs. 6-OHDA+SCMC	-0,1088	-0,2594 to 0,04176	No	ns	0,2634	6-OHDA vs. 6-OHDA+SCMC	-0,6696	-0,8202 to -0,5190	Yes	****	<0,0001
CTR vs. 6-OHDA+SCMC-O	0,4431	0,2925 to 0,5937	Yes	****	<0,0001	6-OHDA vs. 6-OHDA+SCMC-O	-0,1177	-0,2682 to 0,03293	No	ns	0,1948
CTR vs. 6-OHDA+NAC	0,1309	-0,01968 to 0,2815	No	ns	0,1175	6-OHDA vs. 6-OHDA+NAC	-0,4299	-0,5805 to -0,2793	Yes	****	<0,0001
8						8					
CTR vs. 6-OHDA	0,6515	0,5009 to 0,8021	Yes	****	<0,0001						
CTR vs. 6-OHDA+SCMC	-0,09048	-0,2411 to 0,06012	No	ns	0,4479	6-OHDA vs. 6-OHDA+SCMC	-0,7419	-0,8925 to -0,5914	Yes	****	<0,0001
CTR vs. 6-OHDA+SCMC-O	0,5576	0,4070 to 0,7082	Yes	****	<0,0001	6-OHDA vs. 6-OHDA+SCMC-O	-0,0939	-0,2445 to 0,05669	No	ns	0,4098
CTR vs. 6-OHDA+NAC	-0,1241	-0,2747 to 0,02649	No	ns	0,1535	6-OHDA vs. 6-OHDA+NAC	-0,7756	-0,9262 to -0,6250	Yes	****	<0,0001
12						12					
CTR vs. 6-OHDA	0,7499	0,5993 to 0,9005	Yes	****	<0,0001						
CTR vs. 6-OHDA+SCMC	-0,04984	-0,2004 to 0,1007	No	ns	0,8838	6-OHDA vs. 6-OHDA+SCMC	-0,7998	-0,9504 to -0,6492	Yes	****	<0,0001
CTR vs. 6-OHDA+SCMC-O	0,7024	0,5518 to 0,8530	Yes	****	<0,0001	6-OHDA vs. 6-OHDA+SCMC-O	-0,0475	-0,1981 to 0,1031	No	ns	0,9006
CTR vs. 6-OHDA+NAC	-0,01074	-0,1613 to 0,1398	No	ns	0,9996	6-OHDA vs. 6-OHDA+NAC	-0,7607	-0,9113 to -0,6101	Yes	****	<0,0001
16						16					
CTR vs. 6-OHDA	1,09	0,9396 to 1,241	Yes	****	<0,0001						
CTR vs. 6-OHDA+SCMC	0,2038	0,05325 to 0,3544	Yes	**	0,003	6-OHDA vs. 6-OHDA+SCMC	-0,8863	-1,037 to -0,7357	Yes	****	<0,0001
CTR vs. 6-OHDA+SCMC-O	1,025	0,8739 to 1,175	Yes	****	<0,0001	6-OHDA vs. 6-OHDA+SCMC-O	-0,06563	-0,2162 to 0,08496	No	ns	0,7366
CTR vs. 6-OHDA+NAC	0,09099	-0,05960 to 0,2416	No	ns	0,4421	6-OHDA vs. 6-OHDA+NAC	-0,9992	-1,150 to -0,8486	Yes	****	<0,0001
24						24					
CTR vs. 6-OHDA	1,349	1,199 to 1,500	Yes	****	<0,0001						
CTR vs. 6-OHDA+SCMC	0,78	0,6294 to 0,9306	Yes	****	<0,0001	6-OHDA vs. 6-OHDA+SCMC	-0,5695	-0,7201 to -0,4189	Yes	****	<0,0001
CTR vs. 6-OHDA+SCMC-O	1,311	1,161 to 1,462	Yes	****	<0,0001	6-OHDA vs. 6-OHDA+SCMC-O	-0,03813	-0,1887 to 0,1125	No	ns	0,9529
CTR vs. 6-OHDA+NAC	0,6098	0,4592 to 0,7604	Yes	****	<0,0001	6-OHDA vs. 6-OHDA+NAC	-0,7397	-0,8903 to -0,5891	Yes	****	<0,0001

Table S1. Significance data relative to TMRM analyses (**Figure 8**) at different time points.
